# A temporal switch model for estimating transcriptional activity in gene expression

**DOI:** 10.1093/bioinformatics/btt111

**Published:** 2013-03-11

**Authors:** Dafyd J. Jenkins, Bärbel Finkenstädt, David A. Rand

**Affiliations:** ^1^Warwick Systems Biology Centre and ^2^Department of Statistics, University of Warwick, Coventry CV4 7AL, UK

## Abstract

**Motivation:** The analysis and mechanistic modelling of time series gene expression data provided by techniques such as microarrays, NanoString, reverse transcription–polymerase chain reaction and advanced sequencing are invaluable for developing an understanding of the variation in key biological processes. We address this by proposing the estimation of a flexible dynamic model, which decouples temporal synthesis and degradation of mRNA and, hence, allows for transcriptional activity to switch between different states.

**Results:** The model is flexible enough to capture a variety of observed transcriptional dynamics, including oscillatory behaviour, in a way that is compatible with the demands imposed by the quality, time-resolution and quantity of the data. We show that the timing and number of switch events in transcriptional activity can be estimated alongside individual gene mRNA stability with the help of a Bayesian reversible jump Markov chain Monte Carlo algorithm. To demonstrate the methodology, we focus on modelling the wild-type behaviour of a selection of 200 circadian genes of the model plant *Arabidopsis thaliana*. The results support the idea that using a mechanistic model to identify transcriptional switch points is likely to strongly contribute to efforts in elucidating and understanding key biological processes, such as transcription and degradation.

**Contact:** B.F.Finkenstadt@Warwick.ac.uk

**Supplementary information:** Supplementary data are available at *Bioinformatics* online.

## 1 INTRODUCTION

One of the archetypal challenges of systems biology is the task of uncovering the network of interactions between genes and proteins using data such as that coming from high-throughput genome-wide technologies or multi-parameter imaging. Time series gene expression data from techniques such as NanoString, reverse transcription–polymerase chain reaction, microarrays or advanced sequencing are particularly valuable for addressing such tasks especially if the system can be perturbed in an informative way. Such data can also be used to get genome-wide understanding of the variation in key biological processes, such as transcription and degradation. In many cases, one is concerned with better-understood systems, such as the circadian clock or cell cycle, where relatively sophisticated models exist. In these cases, it is of interest to uncover both new connections and deeper details of the regulatory interactions. However, when studying systems where there is a much lower density of understanding, one is relatively satisfied with gaining information on the likelihood of the existence of a regulatory interaction or the importance of a regulatory mechanism. Almost all examples studying the response dynamics when systems are subjected to perturbations, such as drug dosing (Eisen *et al.*, 1998) or stress (Windram *et al.*, 2012), or where the progression of disease is studied (Calvano *et al.*, 2005) fall into this latter category.

Analysis of genome-wide time series gene expression data typically involves a number of tasks to parse the time series into groups using various criteria, identify differential expression, select smaller sets of genes for comparative analysis, identify molecular signatures and common regulatory elements, sort the data to identify processes active at certain times and apply network reconstruction algorithms to identify regulatory interactions. One is, therefore, interested in computational approaches to check the similarity or difference in time series expression between genes and conditions. Many techniques for analysing expression profiles have been used [see Androulakis *et al.* (2007) and Bar-Joseph (2004) for overviews], such as hidden Markov models (Schliep *et al.*, 2004; Yoneya and Mamitsuka, 2007), spline functions (Bar-Joseph *et al.*, 2003; Grün *et al.*, 2012) and clustering (Heard *et al.*, 2005; Kiddle *et al.*, 2010). Unfortunately, however, it is relatively rare that, in terms of specific molecular mechanisms, there is much common regulation found across the clusters produced by such methods. This is perhaps less surprising when one notes that the temporal profile of gene expression depends on several processes, such as transcription, degradation and splicing, and that similar profiles can be produced from different combinations of these processes. In particular, the amount of mRNA for a particular gene is the balance between its synthesis and degradation at any point in time. It would, therefore, be helpful if one could identify the effect of these different processes from the data.

This requires the development of algorithms to provide more mechanistic insight by combining time-course expression data with parametric models of gene expression, and there has been some progress in this direction. Relatively sophisticated methods often using stochastic simulation have been developed for extracting parameter estimates from high-resolution time series data (Golightly and Wilkinson, 2011; Komorowski *et al.*, 2009; Toni *et al.*, 2009). However, these approaches are geared towards modelling the intrinsic noise associated with the birth and death processes of molecules in single cells and are less suitable for aggregate mRNA data arising from microarray and sequencing experiments. Recently, a flexible parametric model for the response of gene expression to environmental perturbations has been introduced in Chechik and Koller (2009) and can be used in this context. Applying it to gene expression time courses in *Saccharomyces cerevisiae* after diverse environmental perturbations they show that their model, which is based on the product of two sigmoid functions and thus can capture exactly two transitions in the response dynamics, constitutes an improvement over other general functional forms, i.e. polynomials. However, although the magnitude and the timing of the response arise as meaningful parameters, their model is not directly connected to mechanism and is still not general enough to explain a wider range of possible dynamic pattern observed in gene expression, including oscillations.

Consequently, there is a need not only to decouple transcriptional from degradation processes but also to model general forms of transcription in a way that is compatible with the demands imposed by the quality, time-resolution and quantity of the data. In particular, the ability to handle an arbitrary number of transitions where the transcription rate is changed and to infer the number and types of these transitions from such data would clearly be an extremely desirable feature. In this article, we propose an ordinary differential equation model (ODE) model that addresses these issues and at the same time can be effectively fitted to data with sufficient computational efficiency to enable one to handle many genes. It is based on a simple dynamical model of mRNA synthesis and degradation, where transcriptional activity can ‘switch’ between an arbitrary number of states. The timing and number of transitions, or ‘switches’, can be estimated efficiently alongside mRNA stability with the help of a reversible jump Markov chain Monte Carlo (RJMCMC) estimation algorithm (Green, 1995). Multiple change-point or switching models have previously been applied to biological systems, such as inferring transcription factor interactions (Opper and Sanguinetti, 2010; Sanguinetti *et al.*, 2009), modelling negative feedback in circadian clocks (Aase and Ruoff, 2008) and reconstructing unobserved gene expression dynamics (Finkenstädt *et al.*, 2008; Harper *et al.*, 2011). However, these models have so far only supported binary expression dynamics, which are not general enough to capture expression dynamics with multiple steady-state expression levels.

The structure of the article is as follows. We first introduce the modelling approach and estimation algorithm. The performance of the algorithm has been studied extensively for artificial data (Supplementary Section ‘Simulation study’). To demonstrate the methodology and its potential further uses, we focus on modelling the wild-type behaviour of a set of 200 chosen oscillatory expressed genes of the model plant *Arabidopsis thaliana*. The approach allows us to investigate whether genes with similar switch event times also have correlated promoter motifs. Furthermore, we introduce a Bayesian hierarchical approach to pool data from several experiments and present results for estimation of mRNA stability. The example datasets consist of time series from three experiments (called E1, E2 and E3) of varying timescales and sampling regimes under some mock treatment conditions (see Supplementary Fig. S1 for examples). Each experiment originally consists of >30 000 probes [Sclep *et al.* (2007); www.catma.org], which map >25 000 genes from the TAIR9 genome annotation [Lamesch *et al.* (2012); www.arabidopsis.org]. Here, we focus on a subset of 200 oscillatory genes (chosen according to their correlation to a sine function for the expression data from E1). The set includes a number of ‘core’ circadian clock genes (such as LHY, AT1G01060; CCA1, AT2G46830; TOC1, AT5G61380). A list of the 200 genes can be found in Supplementary Table S1.

## 2 A MULTI-SWITCH MODEL AND ITS INFERENCE

We assume that the aggregate dynamics of mRNA over a population of cells can generally be described by a piecewise linear ODE model where because of transcriptional regulatory processes, the transcriptional rate of a gene changes (‘switches’) from 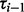 to another rate 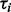 at time point 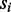

(1)
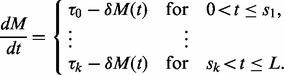



Here, 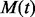 denotes mRNA concentration at time 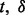 is the rate at which mRNA is degraded and *L* is the length of the time interval over which gene expression is observed. An increase in the transcription rate 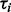 from the previous regime can be interpreted as an ‘on-switch’, whereas an ‘off-switch’ is associated with a decrease of transcriptional activity. However, we note that the expression might not be fully turned off, and that there may be more than just two states. We will refer to the model in (1) as *switch model*. Neither the location of the *switch-times* 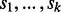 nor the number of *switches k* is known and need to be estimated along with the kinetic parameters of the model. Solving the linear ODE for each linear regime and iteratively inserting the final state of a previous regime as initial condition of the next regime one can derive the following general solution
(2)
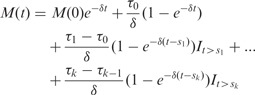

for an initial condition 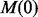, where 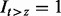 if 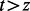 is an indicator function. Note that by setting

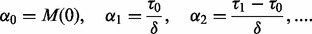

and

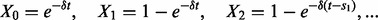



Equation (2) is a linear model
(3)
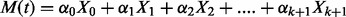

for given degradation rate 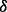 and switch-times 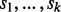. The dimension of the model is determined by the number of switches *k*. The case of no interior switch points corresponds to 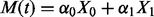, that is the solution of a single linear ODE from an initial condition 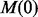, whereas each additional switch-point adds another additive term allowing for convergence to a new equilibrium. Inference is carried out assuming that the ‘true’ model is unknown but comes from a class of models 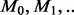 where 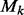 denotes the model with *k* switching points. Using the notation of (3), each model 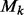 is associated with a parameter vector 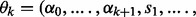 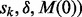, the dimension of which changes with the model. Inference about *k* and 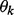 is based on the target distribution that is the joint posterior 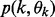. As this is a case of changing model dimension, we shall generate samples from the joint posterior using reversible jump Metropolis Hastings (Green, 1995).

Let 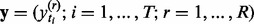 denote the gene expression data for a particular gene where *R* denotes the number of replicate time series, each with *T* observations for a given experimental setting. Note that the notation that each replicate has *T* observations is only used for simplicity. It will be obvious how to allow for a different number of observations per replicate. Assuming that the residuals between the ODE solution (2) and the expression data are i.i.d. normal with mean zero and unknown variance 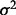, the log likelihood function is
(4)
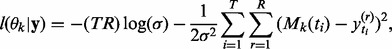

where 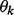 denotes the vector of all unknown parameters, including the initial condition, and 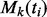 is the solution to the differential equations defined by Equation (2) under model 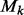.

For given switch-times and degradation rates, we have originally devised a complete Bayesian regression approach to the model in (3) by assigning priors and sample from the conditional posterior (Denison *et al.*, 1998) of the regression coefficients 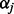. However, analogous to the conclusions of Denison *et al.* (1998) in the context of a spline model, we found that the 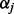 can be calculated by standard least squares regression, which is computationally substantially faster leading to results that, for our purposes, are not distinguishable from the ones obtained from the full Bayesian regression models. Moreover, inference about the actual values of the 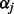 is not directly of interest, as the time series data can only be assumed to be proportional to the concentration 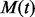, and the values of 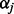 are affected by this scaling.

We use a vague gamma prior for the precision, i.e. 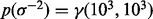 and update the chain for 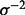 via a Gibbs step as in the usual normal Bayesian regression model. The degradation rate 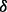 is a parameter shared by all models and ordinary MCMC updating schemes can be applied. Here, we use a vague normal prior for 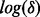 and a random walk Metropolis updating scheme on the log scale. The prior model for *k* can be specified by a Poisson distribution 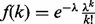 conditioned on 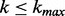 (Green, 1995). Note that by changing 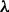, the expected number of switches can be controlled reducing potential model overfitting.

With regard to the switch points, we adapted the reversible jump specifications of the algorithms used in Green (1995) and Denison *et al.* (1998) to our model. We assume that the prior switch-time positions 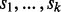 are uniformly distributed on [0, *L*] and classify three possible moves:

- movement of a randomly chosen existing switch-point 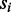 with probability 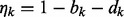;
- addition of a switch with probability 
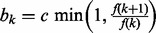
; and
- deletion of a switch with probability 
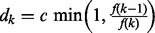

for some constant 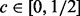. For 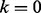, we set 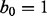 and 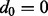. The acceptance probability for the moves follows the general rule (Denison *et al.*, 1998; Green, 1995)
(5)
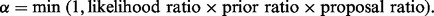



For the position change in (i), we randomly chose a switch-time 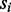 from the *k* existing switches, and a candidate value 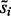 is drawn uniformly on 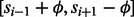, where 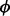 is a fixed minimum time between switch-times. The acceptance probability (5) for this move is

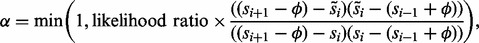

where likelihood ratio here generally refers to the ratio of likelihood of proposed new values of parameters divided by the current likelihood. For move (ii), addition of a switch, we propose a new switch-time 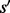 uniformly on 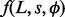, the support of *L* given the constraints imposed by 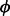 on switch-times *s*. The proposed value will lie in some interval 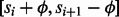. The prior ratio is then

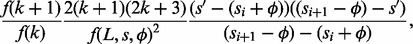

the proposal ratio is 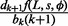 and the acceptance probability for a suggested switch is computed by inserting these into (5). Finally, for move (iii) a switch is chosen randomly from the set of existing switches, and the acceptance probability for this move has the same form as for move (ii) with all ratios inverted.

**Fig. 1.**
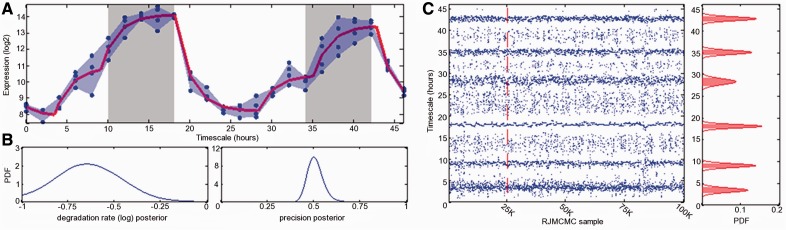
Output from the RJMCMC for the gene LHY in E1. (**A**) The expression data (individual samples shown by 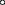 with shading between the extreme samples at each time point). E1 data have a 16:8-h light–dark cycle, with the light periods starting at 18 and 42 h and the dark periods starting at 10 and 34 h. Grey-shaded regions indicate periods of no light. The red line shows the fitted piecewise linear ODE model for mean posterior parameter values. Time in hours is on the *x*-axis, and mRNA expression in log-scale is on the *y*-axis. (**B**) The posterior distributions for the sampled degradation rate (left) and precision (shown here in terms of σ) (right). (**C**) The sampled switch-times (left) and the posterior distributions (right) for the six estimated switches, *s*, and the shaded regions show 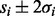. 100 K RJMCMC iterations are generated, and the end of the burn-in period is indicated by the dashed red line at 25 K iterations on (C)

Figure 1 shows the fit of the switch model to E1 data for the core clock gene LHY (AT1G01060). The algorithm estimates a mean half-life of 1.3 h for LHY mRNA and identifies a total of six switches, which consist of three periodically recurring switches per day. LHY is a core regulating component of the *A.thaliana* clock, and it has been shown to be induced before dusk and has a peak of expression at dawn (Schaffer *et al.*, 1998). Around dawn, other clock components repress the expression of LHY resulting in rhythmic expression (Pokhilko *et al.*, 2012). The E1 data have a 16:8 h light–dark cycle. The estimated periodic switches show the initial gene induction several hours before dusk and repression at or shortly after dawn.

This example clearly shows that the model is able to identify asymmetric oscillations resulting from unequal length of on and off times and switches that cause additional modes or ‘shoulders’ in the cyclic patterns. The traces of the RJMCMC algorithm for the switches and their times are plotted in Figure 1C. We have summarized the posterior results by the marginal distribution of all accepted switch-times by fitting a Gaussian mixture model to a function estimated by a non-parametric kernel density. From this, all local maxima are identified and approximated by fitting a mixture of Gaussians. We found this to work well in simulation studies, but note that other parametric or non-parametric approaches could be applied here. Each mode represents a possible switch, and the location is summarized by the mean and the corresponding two-sigma band (shown in Fig. 1C). The latter could be taken as an indicator of the ‘strength’ of a switch. It should, however, be noted that by summarizing the marginal distribution over all accepted switch-times, we are averaging our posterior information over all models entertained by the RJMCMC algorithm. Although we do not follow this in detail, here, we note that it may be useful to investigate the MC traces in more detail for correlation between models.

Convergence for gene expression datasets from E1 is usually achieved after 5 K iterations (Supplementary Fig. S2), and the posterior densities can be estimated from the RJMCMC traces of 75 K iterations, after the first 25 K iterations are discarded as burn-in. The computational time for 100 K iterations was on average 128 s on a 2.8 GHz computer. Fitting this model is thus computationally feasible for thousands of genes and can be easily parallelized.

## 3 RESULTS

To gain a systematic understanding of how the estimation algorithm performs for time series of varying sampling frequencies and noise levels, we generate synthetic datasets, for chosen kinetic parameter values and using data from E1 to obtain realistic sample sizes and noise levels. The full study benchmarking the performance of the model can be found in Supplementary Section ‘Simulation study’. For the synthetic data, we are able to obtain accurate estimates for timing and number of switches and degradation rates under all but the largest noise level (taken as the 95th percentile from the E1 data). We observe that the parameter estimation is invariant of the mode of the switch (increase or decrease transcription rate), that multiple switch points must have at least one observation between them to be reasonably estimated and that a higher sampling frequency is generally more informative for estimation than a larger number of replicate samples. To demonstrate further use of this approach, we now present case studies referring to the 200 example circadian time series.

### 3.1 Correlation of switch-times with promoter motifs

**Fig. 2.**
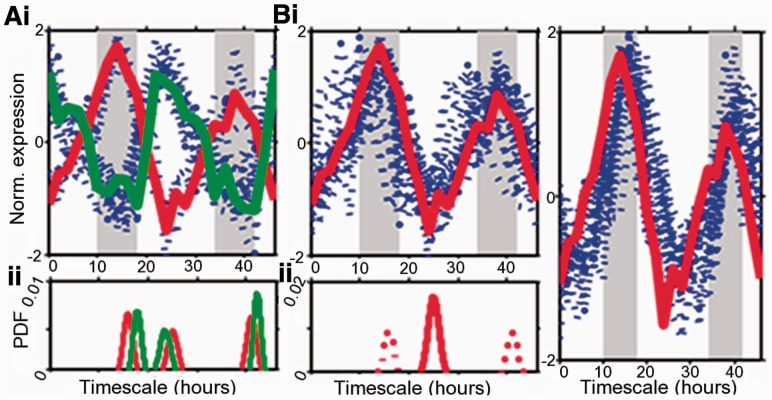
Example clusters using different similarity scores. (**A**) Cluster containing 21 genes based on the switch-time distribution (SD) over the whole time interval: (**i**) normalized expression profiles, highlighted are two example genes with inverted expression dynamics (red = AT1G10740; green = AT1G12845); (**ii**) SD for the two highlighted genes, indicating the three similar switch times. (**B**) Cluster containing 24 genes based on the sum of the individual similarity matrices obtained by separately considering on and off times of the SD: (**i**) normalized expression profiles, with the same gene (AT1G10740) in red; (**ii**) SD for the highlighted gene where the on set is given by the solid line, and the two off sets are given by the dotted line. (**C**) Cluster containing 22 genes, including the same gene (AT1G10740) in red, resulting from using the sum of squared error between normalized expression profiles as similarity score

A common aim of gene expression analysis is to identify potential common regulatory mechanisms between groups of genes through clustering gene expression and enrichment of semantic similarity, such as Gene Ontology (Pesquita *et al.*, 2009), or sequence similarity, such as promoter motif structure (Cooper *et al.*, 2006). Here, we shall use the estimation results from the switch model for the clustering of genes according to similarity in switch-time distributions. Compared with the usual clustering approaches based on similarity of the expression profiles, the basic difference is that we can identify groups of genes that change transcriptional activity around the same times irrespective of mRNA stability and whether such regulation is up or down. Our clustering is based on the similarity matrix whose entries quantify the pairwise distance between the estimated posterior marginal distributions of switch-times (SD) of the genes. Figure 2 shows an example cluster from the 200 circadian genes where we used the symmetric Kullback–Leibler (KL) distance (Kullback and Leibler, 1951). The KL distance is a common choice for computing distances between probability densities, but we note that other distance measures could be implemented in a straightforward way.

Clustering is performed in all cases applying the affinity propagation algorithm (Frey and Dueck, 2007) to the similarity matrix. Multiple similarity matrices can be linearly combined, allowing SDs from multiple experiments to be combined. One can also focus the clustering on subsets of the domain of the SD. If the domain is equal to the total length of observational time, then genes with the same switch-times are clustered together irrespective of whether they are on or off switches. As the genes are all circadian here, the corresponding expression profiles of the genes in that cluster seem to be either in the same or in the opposite ‘phase’ (Fig. 2A). If the distance measure is applied separately to the on and off times, depending on whether transcription is increased or decreased, then two similarity matrices can be defined for each gene pair. Applying the clustering to the sum of the two resulting similarity matrices gives clusters where the expression patterns are in phase (Fig. 2B). The difference from clustering according to overall similarity of the expression profile is visible in Figure 2C where the red-highlighted example gene is now in a group of more highly correlated expression profiles, but the timing of its switches is visibly earlier than for most other genes in that cluster.

A commonly explored hypothesis is that correlated gene expression patterns will also have correlated promoter structure and regulation mechanisms. In practice, such correlations have yet to be confirmed. By combining our analysis of switch-time similarity with promoter motif data we ask whether our approach can shed more light on this issue. We investigate how frequently certain listed motifs are encountered in clusters of genes. If a motif has a high frequency for the genes in a given cluster then it is more likely that the corresponding transcription factor binding sites are key for the regulation of those genes. This could give us an indication of which genes may be turned on or off by the same transcription factors. Using position-specific scoring matrix (PSSM) data from the TRANSFAC (Matys *et al.*, 2006) and PLACE (Higo *et al.*,1999) databases, 25 plant motifs were selected, where each motif is present in at least 50 of the 200 circadian gene subset, identified using APPLES (Baxter *et al.*, 2012) (see Supplementary Tables S2 and S3 for motifs). These motifs can be grouped by sequence similarity into three broad classes of promoter motifs, and a similarity matrix is generated from co-occurrence of motifs between each pair of genes, which can then be linearly combined with each of the three similarity scores and clustered.

One could obtain a clustering based on motif co-occurrence alone (Supplementary Fig. S3A), which does not yield any temporal correlation in the resulting expression profiles (Supplementary Fig. S4). On the other hand, clustering only the expression time series (Supplementary Figs S5–S7) brings up some correlated promoter structure when using switch-times, rather than overall expression (Supplementary Fig. S3B). However, the approach is most informative when both the motif co-occurrence and time series information are combined in that we are able to identify strong correlations in promoter structure together with temporal separation of profiles between clusters.

**Fig. 3.**
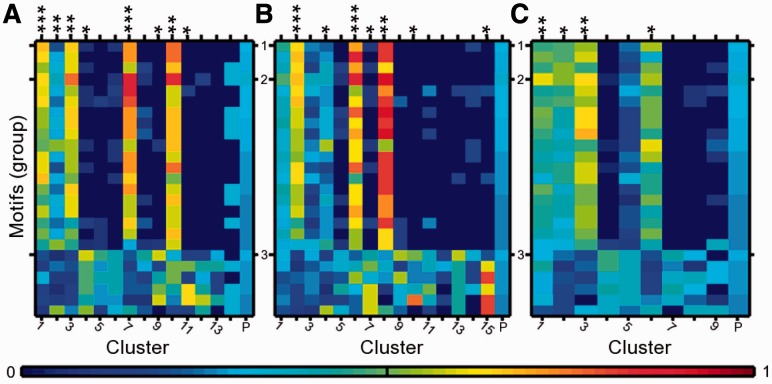
Heatmaps of proportion of motifs present in gene clusters using different similarity measures. Motifs are aligned on *y*-axis and grouped into three classes by sequence similarity. Each column gives the proportion of motifs in a gene cluster. (**A**) Motif co-occurrence is combined with the switch-time distribution (SD) similarity matrix over the whole time interval, (**B**) motif co-occurrence is combined with the SD similarity separately over on and off times. (**C**) Motif co-occurrence is combined with similarity of the overall expression profiles. Clusters are assigned significance by the hypergeometric test with false discovery rate correction on the cluster motif proportions against the population motif proportions; 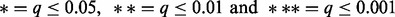. Cluster ‘P’ shows the population motif proportions

Figure 3 shows heatmaps for the proportions of each motif present in each of the clusters resulting from combining the motif co-occurrence similarity matrix with the similarity score of either the switch-times or the overall expression profiles. The last column (P) gives the proportion of each motif in the overall population of the 200 circadian genes. It can clearly be seen that clusters based on the whole switch-time distribution together with motif co-occurrence show a very high proportion of some motifs in several clusters. For instance, in Cluster 1, all motifs from Group 1 and 2 (19 of 25 motifs) are present in 40% or more of the genes, and in Cluster 7, 11 motifs from Groups 1 and 2 are present in >70% of genes, whereas the population mean motif proportion is only 28% (Fig. 3A). Clusters 1 and 7 contain a number of significantly overrepresented promoter motifs, with 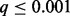 (denoted by triple asterisk in Fig. 3) using the hypergeometric test and corrected with a false discovery rate of 5% (Benjamini and Hochberg, 1995). Eight clusters contain significantly overrepresented motifs and a clear separation of genes containing motifs from Groups 1, 2 and 3. A similar result is observed using the additively combined separate on and off components and motif co-occurrence for clustering (Fig. 3B), which, naturally, results in a slightly larger number of clusters. Comparing with clustering based on expression profiles together with motif co-occurrence, we find that the proportion of motifs in clusters is generally smaller and fewer significant clusters are observed (Fig. 3C). Plots of the expression profiles separated into clusters are shown in Supplementary Figures S8–S10. Cluster membership and motif proportions for the combined similarity measure clusters are given in Supplementary Tables S4–S7.

This example makes it apparent that clustering based on switch-times combined with motif co-occurrence is useful in identifying correlation between gene expression and promoter structure and, therefore, also in identifying potential regulatory interactions. Motif instance data are often noisy because of redundancy and degeneracy in PSSMs, and in genome-scale expression data sets many genes may share expression profiles, but not regulatory dynamics. By incorporating switch-times with motif data we can link specific temporal events in transcription with specific promoter structures.

### 3.2 Hierarchical modelling of multiple time series

Provided that the mRNA process exhibits non–steady-state behaviour, the use of the switch model allows for inference on mRNA degradation rates for many genes without having to resort to additional experiments that are specifically targeted at transcriptional inhibition. Although it is straightforward to obtain posterior distributions from one experiment, it may also be of interest to pool the information from several experiments allowing for the possibility that, for a given gene, the mRNA degradation rate across the experiments should be similar but allow for variation because of the setting of the experiment (different laboratories, techniques, mock treatments and time spans). We also wish to incorporate informative prior information from the study by Narsai *et al.* (2007), which gives estimates of mRNA degradation rates of >13 000 *A.thaliana* genes while noting that use of such data is problematic because, by its nature, there is no independent validation of the results of such studies, and their experimental conditions are different from ours. We address this in the following Bayesian hierarchical modelling framework.

Let 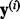 denote the time series data (including replicates) for a particular gene in experiment *i* and 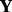 the pooled data for that gene over *N* experiments. Bayesian hierarchical modelling (Gamerman and Lopes, 2006) provides a natural framework to account for the fact that parameters may be similar but are subject to stochastic variability between experiments. Assuming experiments are independent, the full log likelihood is
(6)
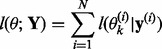

where 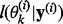 is the log likelihood as specified in (4), and each 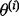 is a vector of varying dimension 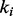 containing all model parameters for experiment *i*. In a Bayesian hierarchical model, we assume that parameters or subsets of the parameters are similar but subject to some stochastic variation in the sense that they are realizations of the same probability distribution whose parameters we wish to identify. Here, we assume that the mRNA degradation rate for a gene across the experiments comes from the same distribution 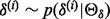, which is characterized by the parameter vector 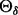. The latter thus quantifies the mean value and variability of the degradation rate across the experiments. It should be noted that with respect to the switch-times we maintain a non-hierarchical structure, as regulatory switches may generally occur at different times for each of the experiments given that the initial entrainment of the clock may have varied across the experiment, and the different light/dark input for each experiment may have caused phase changes. However, the hierarchical approach can be used for scenarios where there is reason to assume that switch-times are similar between experiments. Inference is achieved by formulating an appropriate MCMC algorithm that samples from 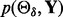 (Gamerman and Lopes, 2006). In practice, we specified 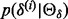 by a Gamma distribution parameterized to have mean 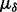 and variance 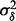. We assigned a Gamma prior distribution to the mean 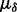 and an exponential distribution to the coefficient of variation (CV) 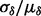. The CV is used, as the mean value is close to 0; therefore, it is a more robust parameter for sampling 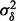. We derive informative priors from degradation rates from >13 K *A.thaliana* genes from Narsai *et al.* (2007):

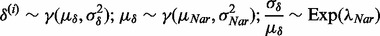

where 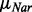 and 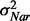 are estimated from the mean degradation rates in the dataset, and 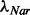 is taken to be the 95% percentile of the CV distribution from the data. All models use the same informative population-level prior distributions, rather than the gene-specific estimates because of large potential differences between experimental protocols. The approach taken by Narsai *et al.* was to fit exponential decay least squares regression models to microarray data collected at six time points after the transcriptional inhibitor actinomycin D was added. Although this is conceptually a straightforward approach for estimating degradation rates, it is neither clear whether the method completely inhibits transcription nor whether its invasiveness has an effect on the degradation processes themselves. Therefore, a strong gene-specific prior derived from the Narsai *et al.* estimates could not be justified.

**Fig. 4.**
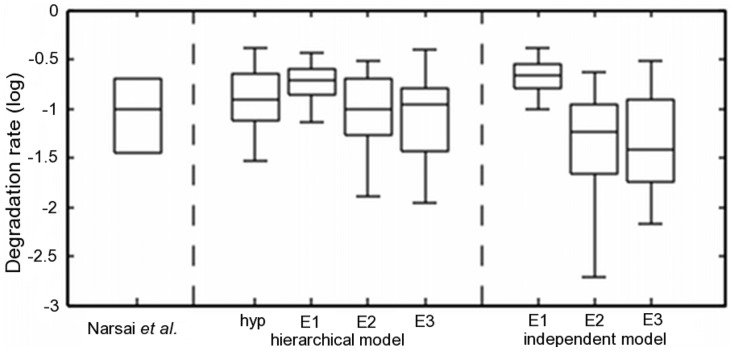
Box plots of degradation rate estimates for gene LHY. Degradation rate estimates visualized for the gene LHY obtained experimentally (left), hierarchical model; hyperdistribution and the three individual experiment estimates (centre) and estimates from the independent models for each individual experiment (right). Data for experimentally obtained degradation rate estimates are taken from Narsai *et al.* (2007). Box plots for model estimates show 25th–75th percentiles and whiskers at 5th and 95th percentiles, whereas the Narsai *et al.* box plot shows mean 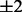 standard error

Figure 4 shows all estimates of the mRNA degradation rate of the core clock gene LHY from Narsai *et al.*, the hierarchical model and, for comparison, the non-hierarchical (independent) model. The Narsai *et al.* estimate has an approximate range in half-life of 1.5–2.25 h, and our posterior estimates are broadly in a similar range. The estimated joint distribution summarizes the variability of the three experiments and provides a theoretically rigorous summary statistic of the degradation rate (which cannot be achieved by averaging over the independent results). Estimates from the individual models show a range of estimates from 1.3 to >2 h. Results for E2 and E3 are more variable probably because they cover shorter timescales of 17.5 and 6 h, whereas E1 covering two circadian cycles provides more precise estimates despite smaller sampling frequency. An interesting observation is the difference between the E1 estimate and E2, E3 and Narsai *et al.* estimates. There are a number of potential reasons for the difference, given the experiments were performed over different time intervals and in different laboratories. However, it may also be related to the light conditions, as E1 is the only experiment incorporating two 8 h dark periods. Light is a key driver in the *A.thaliana* circadian clock, and a recent study has suggested a light-specific degradation rate for CCA1, a core partner of LHY (Yakir *et al.*, 2007), and a current model for the *A.thaliana* clock uses different degradation rates for the LHY/CCA1 component in light and dark (Pokhilko *et al.*, 2012). This result complements the hypothesis that light plays a role in mRNA degradation in at least some core circadian clock components. A possible extension to the model could include light/dark cycles, for which separate degradation rates are assumed.

For further comparison with the Narsai *et al.* estimates, we grouped all genes for which there was an estimate available in their dataset (136 of the 200 genes) into five broad mRNA stability groups based on half-life, as used by Narsai *et al.* (0–1, 1–3, 3–6, 6–12 and 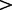
12 h). We find that 32% (43 of 136 genes) have a similar hyperdistribution estimate using our approach to the Narsai *et al.* study, and agreement between the three individual estimates and Narsai *et al.* ranges from 34% up to 36% (Supplementary Table S8). However, despite this overlap, there is also considerable variability in degradation rates between the experiments, which may be natural variability or because of the experiments carried out under different conditions.

## 4 DISCUSSION

The aim of this article is to present a novel approach for identifying timing of transcriptional activity from time series mRNA expression data. The model introduced here consists of a piecewise linear simple ODE model of mRNA dynamics, which can be fitted efficiently with a RJMCMC sampler to estimate gene-specific parameters, i.e. mRNA stability and number and times of switches in transcriptional activity. Estimation and performance of the algorithm is investigated for synthetic data of varying sampling frequencies and noise levels in a simulation study. With the example of time series microarray data from 200 circadian genes, further directions are explored exploiting different aspects of the model output. Namely, using the timing of the switches as a basis for clustering, which, when combined with promoter motif data, seems to identify more significant groups of motifs than simple profile clustering with promoter motif data, potentially implying a stronger correlation with regulatory mechanisms. We also explore the potential for the estimation of mRNA degradation rates. Usually, degradation rate studies involve treatment with a transcriptional inhibitor, such as actinomycin D, or translational inhibitor, such as cycloheximide. It is not clear whether such inhibition is ever achieved fully and whether such treatments have undesired side-effects on degradation, and may, therefore, impact on estimated rates in unpredictable ways. The model introduced in this study has several advantages over a transcription inhibition study. The primary advantage is that a specific experiment does not have to be designed and performed, often at great cost in time and resources, to produce a suitable dataset for degradation estimation, effectively allowing recycling of existing datasets further increasing their potential scientific value. As only free-running mRNA expression dynamics are required, potential side-effects introduced by using a chemical inhibitor can be avoided. We demonstrate how to pool data from several experiments in a theoretically rigorous way with a Bayesian hierarchical model. Degradation estimates can easily be obtained for suitably resolved time series and can be compared between different experimental conditions. As the number of large high-resolution gene expression time series datasets publicly available is likely to increase with the development of cheaper and faster high-throughput technologies, new methods are required to analyse these data. The model proposed here is mechanistic yet is flexible and rich enough to capture a wide range of expression dynamics observed in mRNA time series data, from steady-state behaviour to oscillatory expression. At the same time, it is simple enough to be estimated with feasible computational time for thousands of genes. Using a mechanistic model to identify transcriptional switch points is likely to strongly contribute to efforts in elucidating and understanding regulatory interactions within transcriptional networks.

## Supplementary Material

Supplementary Data
