# Supplementary material for: A temporal switch model for estimating transcriptional activity in gene expression
Source: Bioinformatics. 2013 Mar 11;29(9):1158–65. doi: 10.1093/bioinformatics/btt111 (PMC3634189; doi:10.1093/bioinformatics/btt111)
Supplement: Supplementary Data [file supp_29_9_1158__index.html]

A temporal switch model for estimating transcriptional activity in gene expression — A temporal switch model for estimating transcriptional activity in gene expression — A temporal switch model for estimating transcriptional activity in gene expression — Supplementary Data 

# A temporal switch model for estimating transcriptional activity in gene expression

## Supplementary Data

files

**Files in this Data Supplement:**

- Supplementary Data - pdf file
- Supplementary Data - xls file
