# Supplementary material for: A temporal switch model for estimating transcriptional activity in gene expression
Source: Bioinformatics. 2013 Mar 11;29(9):1158–65. doi: 10.1093/bioinformatics/btt111 (PMC3634189; doi:10.1093/bioinformatics/btt111)
Supplement: Supplementary Data [file supp_btt111_Switch-Bioinformatics_supp.pdf]

A temporal switch model for estimating transcriptional activity in gene expression
Supplementary Information

Dafyd J. Jenkins, Bärbel Finkenstädt and David A. Rand

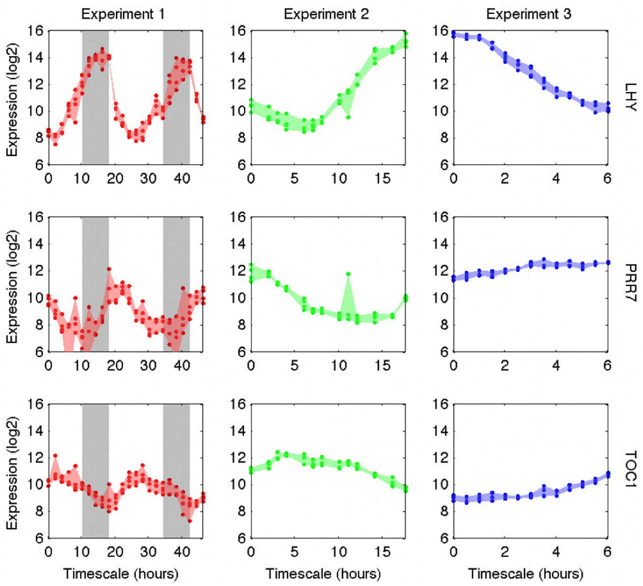

Supplementary Figure 1: Example genes across 3 experiments (E1, E2 and E3). The 3 experiments are wild-type time series consisting of different timescales and sampling frequency from the model organism *Arabidopsis thaliana*. E1 has a 48 hour timescale sampled at 24 time points, E2 has a 17.5 hour timescale sampled at 13 unequally spaced time points. E3 has a 6 hour timescale with 13 time points. For each experiment 4 biological replicates were measured, resulting in a total sample size of 96 for E1, and 52 for E2 and E3. Individual samples are indicated by dots. The coloured shaded areas represent the difference between maximum and minimum replicate measurement at each time point, and the light-dark photoperiods are shown on E1, where grey shaded regions indicate of no light periods. Gene names are given on the right.

A

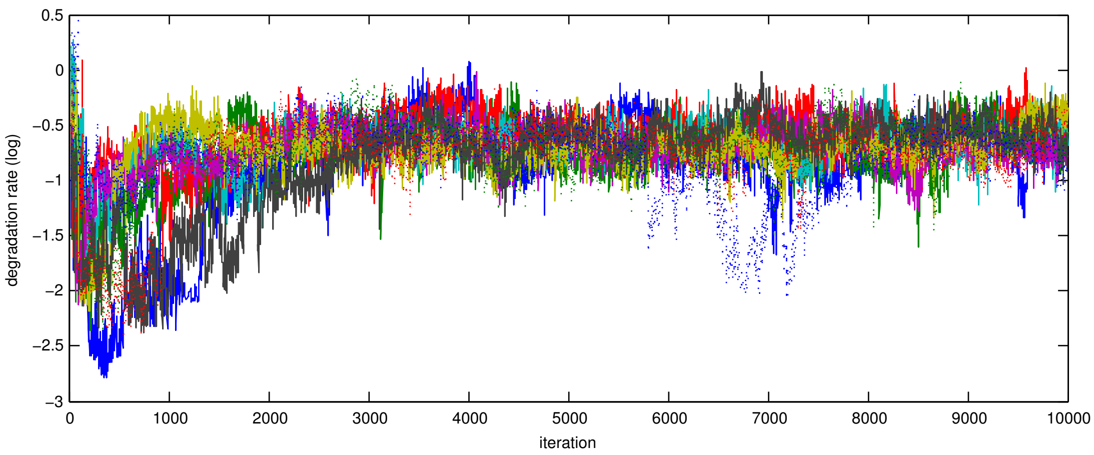

B

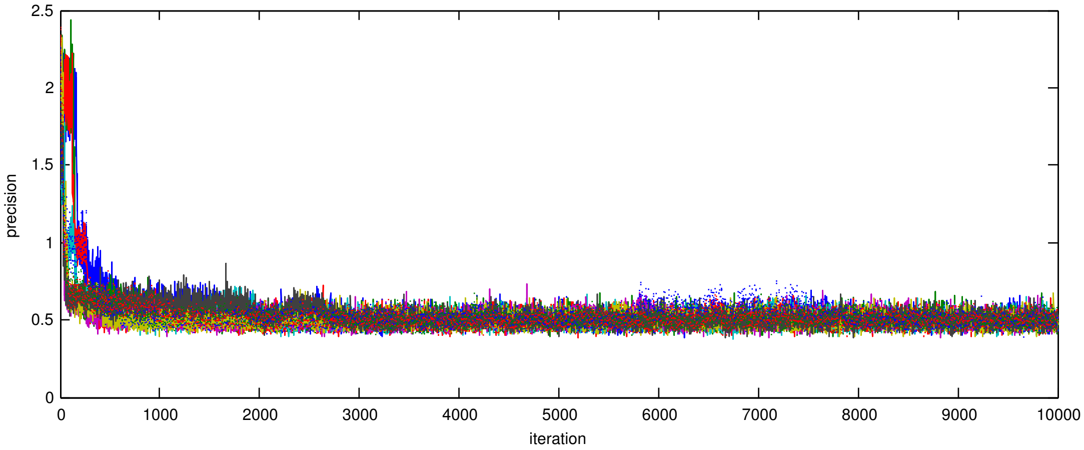

Supplementary Figure 2: Convergence of degradation rate and precision parameters in RJMCMC simulations. Partial chain outputs for 10 independent RJMCMC simulations for the gene LHY. **(A)** shows the first 10,000 iterations from each of the 10 simulations for the degradation rate parameter. Convergence of the chains can be visually identified by around 5000 iterations. **(B)** shows the first 10,000 iterations from each of the 10 simulations for the precision parameter. Convergence of the chains can be visually identified by around 2,000 iterations.

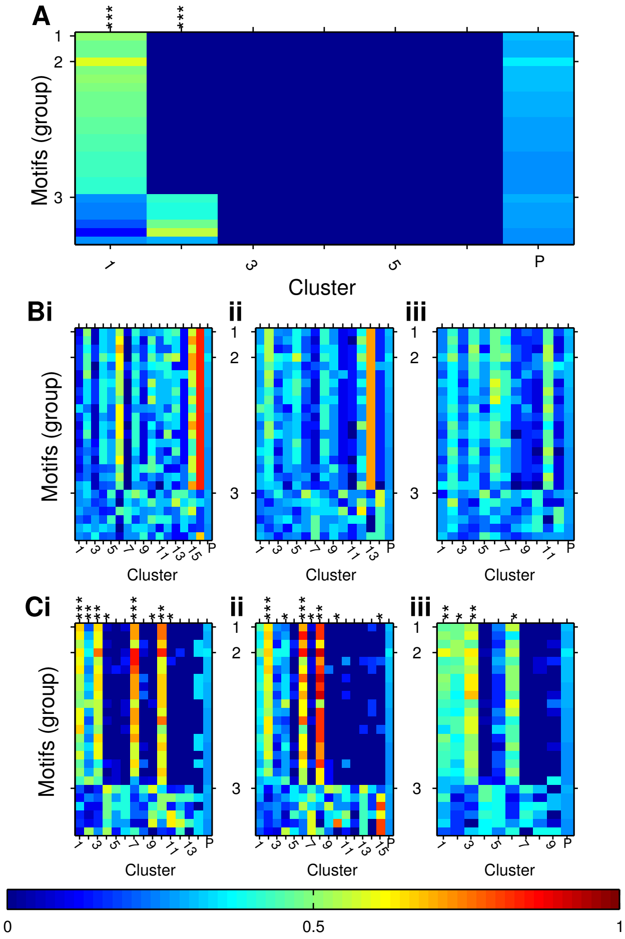

Supplementary Figure 3: Heatmaps of motif proportions in gene clusters identified by different similarity matrix combinations. Motifs are aligned on y-axis and grouped into 3 classes by sequence similarity. Each column gives the proportion of motifs in a gene cluster. **(A)** motif co-occurrence similarity matrix only. **(B)** time series derived similarity matrices only, where similarity was based on **(i)** whole time interval switch distribution (SD), **(ii)** separate on and off SD and **(iii)** expression profile. **(C)** combined similarity matrices from time series and motif co-occurrence, where **(i)** whole time interval SD, **(ii)** separate on and off SD and **(iii)** expression profile. Clusters are assigned significance by the hypergeometric test with false discovery rate correction on the cluster motif proportions against the population motif proportions; $* = q \leq 0.05$, $** = q \leq 0.01$ and $*** = q$.

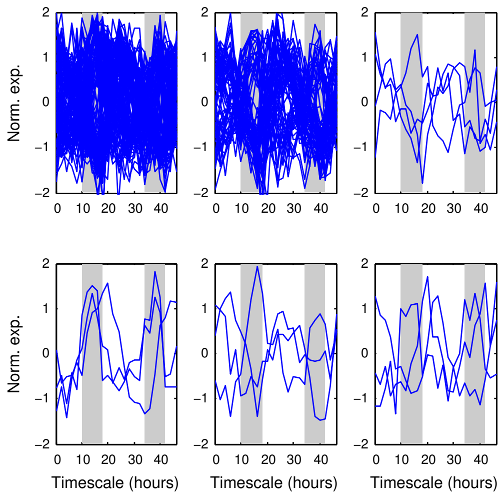

Supplementary Figure 4: Cluster plots for clustering based on the motif co-occurrence similarity matrix only. Plots of the median expression profiles for each member of the 6 clusters obtained using the motif co-occurrence similarity matrix. No temporal correlation can be identified between expression profiles in the clusters. Clusters are ordered from left to right, top to bottom (Cluster 1 top-left, Cluster 6 bottom-right). The light-dark regimes are indicated by the background shading (white = light; grey = dark)

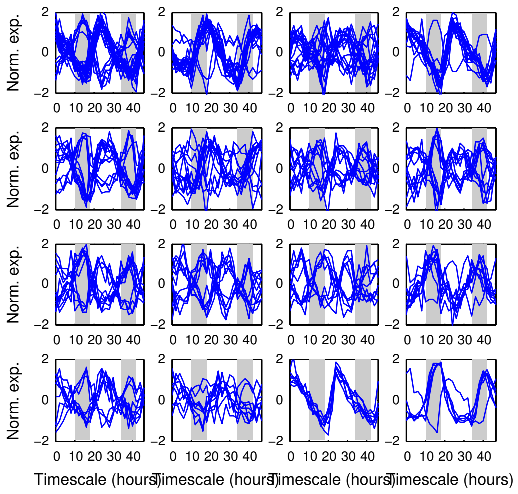

Supplementary Figure 5: Cluster plots for clustering based on the whole time interval SD based similarity matrix only. Plots of the median expression profiles for each member of the 16 clusters obtained using only the SD similarity matrix over the whole time interval. Clusters are ordered from left to right, top to bottom (Cluster 1 top-left, Cluster 16 bottom-centre). The light-dark regimes are indicated by the background shading (white = light; grey = dark)

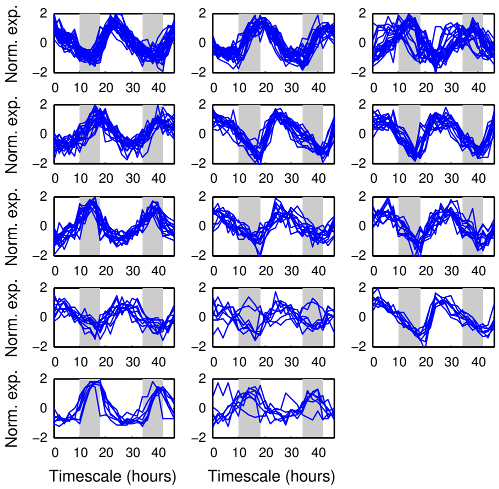

Supplementary Figure 6: Cluster plots for clustering based on the separate on and off SD based similarity matrices only. Plots of the median expression profiles for each member of the 14 clusters obtained using only the linearly combined separate on and off SD similarity matrices . Clusters are ordered from left to right, top to bottom (Cluster 1 top-left, Cluster 14 bottom-right). The light-dark regimes are indicated by the background shading (white = light; grey = dark)

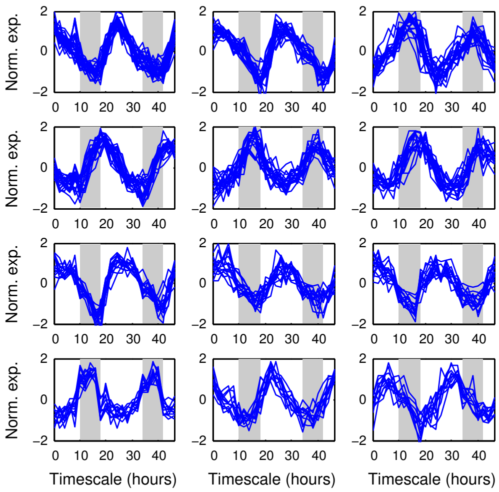

Supplementary Figure 7: Cluster plots for clustering based on the expression profile based similarity matrix only. Plots of the median expression profiles for each member of the 12 clusters obtained using only the expression profile similarity matrix. Clusters are ordered from left to right, top to bottom (Cluster 1 top-left, Cluster 12 bottom-right). The light-dark regimes are indicated by the background shading (white = light; grey = dark)

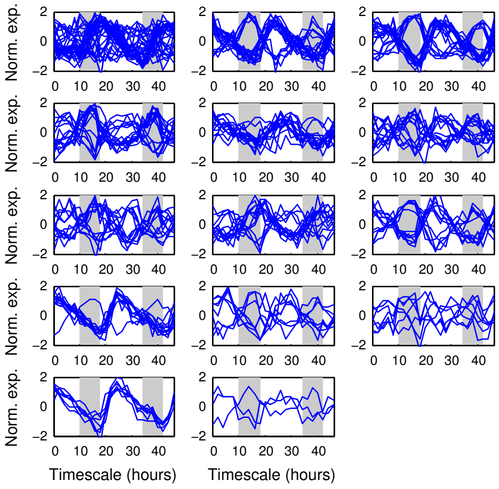

Supplementary Figure 8: Cluster plots for clustering based on the combined whole time interval SD based similarity matrix and the motif co-occurrence similarity matrix. Plots of the median expression profiles for each member of the 14 clusters obtained using the similarity matrix based on the whole time interval SD combined with the motif co-occurrence similarity matrix. Clusters are ordered from left to right, top to bottom (Cluster 1 top-left, Cluster 14 bottom-centre). The light-dark regimes are indicated by the background shading (white = light; grey = dark)

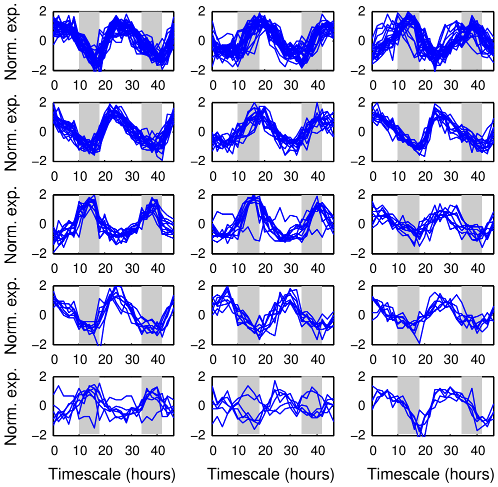

Supplementary Figure 9: Cluster plots for clustering based on the combined separate on and off SD based similarity matrices and the motif co-occurrence similarity matrix. Plots of the median expression profiles for each member of the 15 clusters obtained using the linearly combined separate on and off SD based similarity matrices combined with the motif co-occurrence similarity matrix. Clusters are ordered from left to right, top to bottom (Cluster 1 top-left, Cluster 15 bottom-right). The light-dark regimes are indicated by the background shading (white = light; grey = dark)

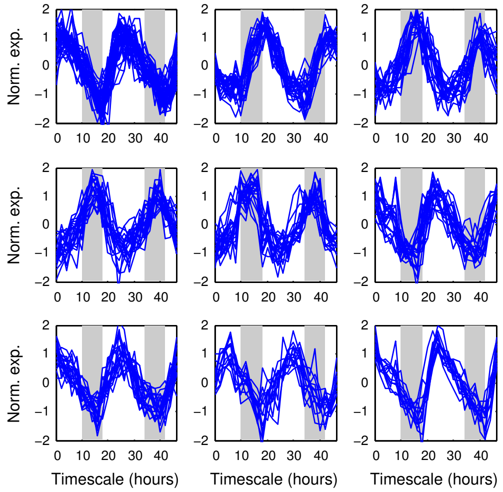

Supplementary Figure 10: Cluster plots for clustering based on the expression profile based similarity matrix combined with motif co-occurrence similarity matrix. Plots of the median expression profiles for each member of the 9 clusters obtained using the expression profile based similarity matrix combined with the motif co-occurrence similarity matrix. Clusters are ordered from left to right, top to bottom (Cluster 1 top-left, Cluster 9 bottom-right). The light-dark regimes are indicated by the background shading (white = light; grey = dark)

Simulation study

In order to gain a systematic understanding of how the estimation algorithm performs for data sets of varying sample sizes, sampling frequencies and noise levels we generate synthetic datasets using known parameter values. In particular, we set $\delta = 0.35$ per h (2h half-life) for degradation and translation rates were taken to change, at selected switch-times, between $\tau = 4.85$ and a less active rate of $\tau = 3.47$, corresponding to steady-state levels of about 14 and 10 expression units of data, respectively. This kind of switch behaviour was visually observed for some genes in E1 under treatment condition and the parameter values stated were obtained from preliminary estimation. We want to characterise the effects of spacing between successive switches, noise level, sampling frequency and the number of biological replicates. To do this it clearly suffices to restrict to the case of two or less switches.

Design

Switch model ODE solutions, given by Equation 1 were sampled at discrete time points and perturbed by Gaussian noise. In order to obtain realistic values for the noise variance we fitted smooth splines for each gene of the full E1 microarray data set and consider noise levels at chosen percentiles of the resulting distribution of residual variances (the noise levels and corresponding mean signal to noise ratios can be found in Supplementary Table 9). In order to study what sampling frequency and number of biological replicates are needed for estimation we impose different 'sampling regimes', each consisting of sampling frequency (every 1, 2 and 4h) and number of replicates (1, 2 and 4) giving a total sample size that ranges from 12 to 196 for a 2-day timescale (as available for E1).

Supplementary Table 9: Noise levels for simulation study

Percentiles	5%	25%	50%	75%	95%
σ	0.18	0.24	0.32	0.44	0.66
SNR	74.75	22.68	39.33	16.93	10.09

We consider 6 different cases (SE1 to SE6) for the dynamics of switch events, ranging in complexity from 0,1 and 2 switches, in the latter case allowing for a different time span (1, 2 and 4h) between the switches (see Supplementary Table 10 for overview). In all cases the switch-time restriction parameter ϕ is set to be equal to the sampling frequency (such that with a sampling frequency of 2 hours, we will only sample switch-times that are 2 hours or more apart). Plots of example time series with different noise distributions can be found in Supplementary Figure 11. Here, we primarily summarize performance for the case of SE2 (single off-switch) and SE6 (2 switches, 4h apart) for a 2h sampling frequency and 4 replicates as in the real data for E1.

Supplementary Table 10: Switch events for simulation study

Regime	No. of switches	Type and time of switch
SE1	0	no switch
SE2	1	off-switch at $s_1 = 24.5h$
SE3	1	on-switch at $s_1 = 24.5h$
SE4	2	off-switch at $s_1 = 24.5h$, on-switch at $s_2 = 25.5h$
SE5	2	off-switch at $s_1 = 24.5h$, on-switch at $s_2 = 26.5h$
SE6	2	off-switch at $s_1 = 24.5h$, on-switch at $s_2 = 28.5h$

Estimation results are generally reported from applying the estimation algorithm to 100 independent samples of synthetic data sets.

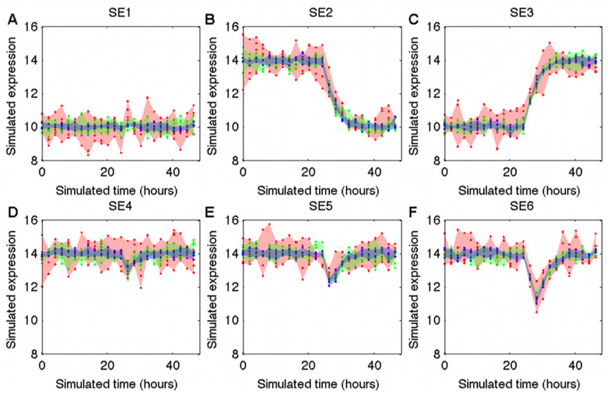

Supplementary Figure 11: Example synthetic time series data (sampling frequency 2h, 4 replicates, as in E1). **A-G**) show example synthetic profiles for SE1 to SE6 with different measurement noise levels, estimated from E1, where blue is the 5th percentile, green is the median level and red is the 95th percentile.

Results

It is clear from the ODE model that the degradation parameter cannot be identified if the observed mRNA process has been in a steady state throughout the observational period (see Supplementary Figure 12A) and that we can only obtain good estimates for genes whose mRNA process exhibits transitional behaviour or switches between different equilibria. Supplementary Figure 13 shows the distribution of the estimated degradation rate δ and a heatmap of the estimated switch-times for increasing noise levels when the algorithm is applied to 100 simulations of synthetic data sets for SE2 and SE6. It seems that the degradation rate can be retrieved reasonably accurately unless the noise level is very high, i.e. 95%. The estimation of the switch-times appears to be robust to noise as both the single and double change-points are estimated with good precision that decreases only marginally for a large noise level.

Can we expect to obtain estimates of reasonable precision for the kind of sample size available to us? Supplementary Figures 14 and 15 show posterior degradation rate and switch-time estimates for different combinations of sampling frequencies and number of replicates of the simulated data sets for the case of SE2 (single off-switch) and SE6 (2 switches, 4h apart), respectively. We note that if the sampling frequency is 4h we cannot obtain any accurate estimates neither of degradation rate (Supplementary Figure 14A) nor switch-time (Supplementary Figure 14B) but estimation is generally improved for the more frequently sampled models. A similar trend is also observed for the model with 2 switches (Supplementary Figure 15) where it appears that the estimation of the second switch suffers from correlation with the degradation rate for the 4h sampling frequency. Again, the quality of the estimation is improved when the number of replicates is increased and/or the data is sampled more frequently and the simulation study confirms that we can expect good precision for data of the size available from E1.

While increasing the sample size, an obvious question is what is preferable: sampling more frequently in time or increasing the number of replicates at given time points (assuming that conditions between replicates are identical)? Here it appears that sampling more frequently achieves more improvement in both estimators. For instance, we can achieve a total sample size of 24 by sampling 2 replicates every 4h, or 1 replicate every 2h. Supplementary Figure 15A shows that the higher sampling frequency allows a reasonable estimate for the degradation rate (sampling regime 2,1), whereas we are not able to produce an estimate with accuracy for the 4h sampling frequency. When the total sample size is doubled to 48, we can sample in 3 ways: every 4h with 4 replicates, every 2h with 2 replicates, or every 1h with just 1 replicate. Supplementary Figure 14A shows that degradation rate estimation is best for the last case.

Further results of the simulation study demonstrate that parameter estimates are of equal quality irrespective of whether the switch is an off or on switch (SE2 or SE3, see Supplementary Figure 12B) and that, in a model with two successive switches (SE4-SE6), one can only detect the second switch and degradation

rate accurately if the time between the two switches is larger than the sampling frequency, i.e. there needs to be at least one observation in the regime between the two switches (see Supplementary Figures 15, 16 17).

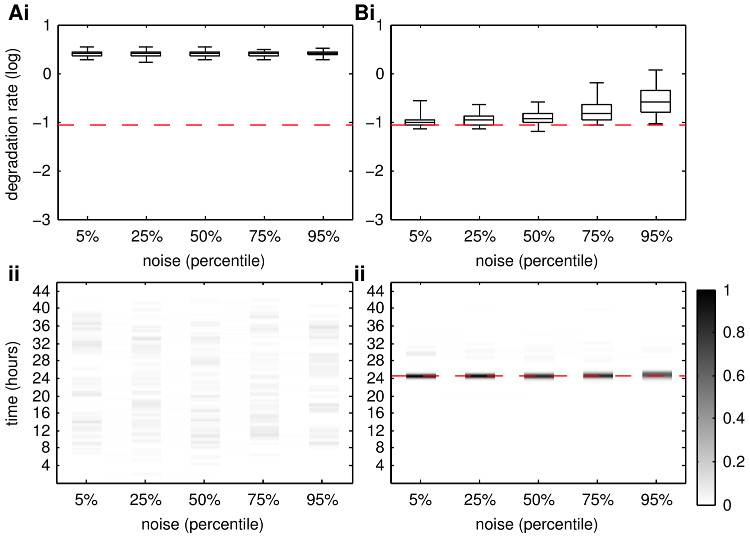

Supplementary Figure 12: Estimation results for 100 model simulations for SE1 and SE3 with different noise levels. (Sampling frequency 2h, 4 replicates). Posterior distributions from (**A**) SE1 (zero switches) and (**B**) SE3 (single on-switch), with 5 levels of measurement noise. i) distribution of estimated degradation rate (log scale) summarised by mean from the posterior distributions obtained from all model fits. Box plots show 25th-75th percentiles and whiskers at 5th and 95th percentiles, and the dashed red line shows the true degradation rate; ii) Heatmap of the Gaussian mixture models fitted to switch-times. Dashed red line shows true switch-times.

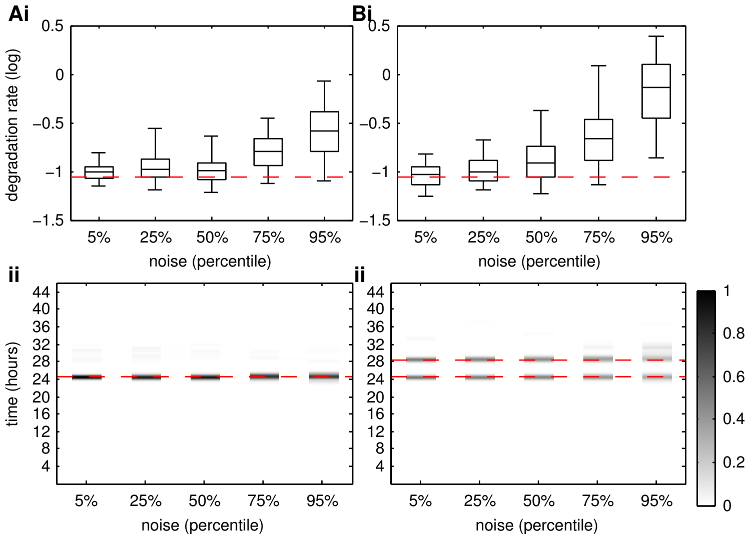

Supplementary Figure 13: Estimation results for 100 model simulations for SE2 and SE6 with different noise levels.(Sampling frequency 2h, 4 replicates). Posterior distributions from (**A**) SE2 (single off-switch) and (**B**) SE6 (2 switches, 4h apart), with 5 levels of measurement noise. i) distribution of estimated degradation rate (log scale) summarized by mean of the posterior distributions obtained from all model fits. Box plots give whiskers at 5th and 95th percentiles. Dashed red line shows true value of degradation rate; ii) Heatmap of the Gaussian mixture models fitted to switch-times. Dashed red lines show true switch-times.

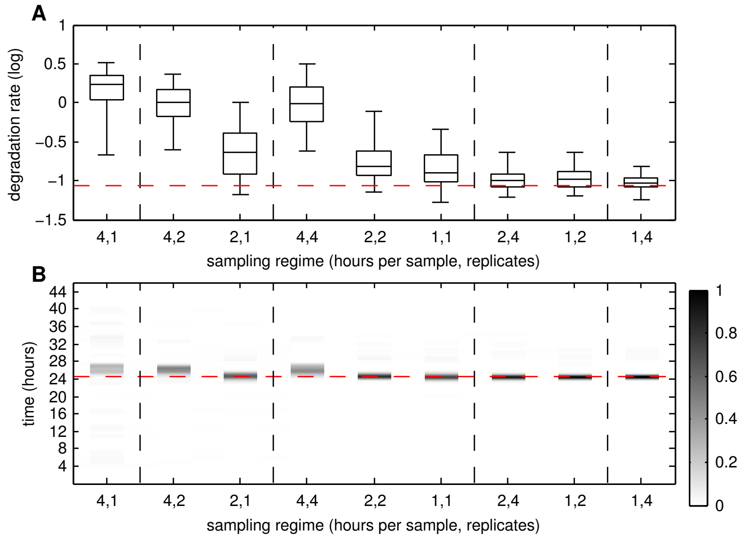

Supplementary Figure 14: Simulation study (sampling frequencies 1, 2 and 4h; Model SE2 with median noise level). Posterior distributions from switch event regime 2. **A**) distribution of estimated degradation rates (log scale) from 100 synthetic data sets for different combinations of (sampling frequency, number of replicates). Dashed red line shows the true degradation rate. **B**) Heatmap of the estimated switch using the Gaussian mixture model. Dashed red line shows true switch-time. Dashed vertical lines separate the sampling regimes by total sample size, in increasing order from 12 (far left) to 196 (far right).

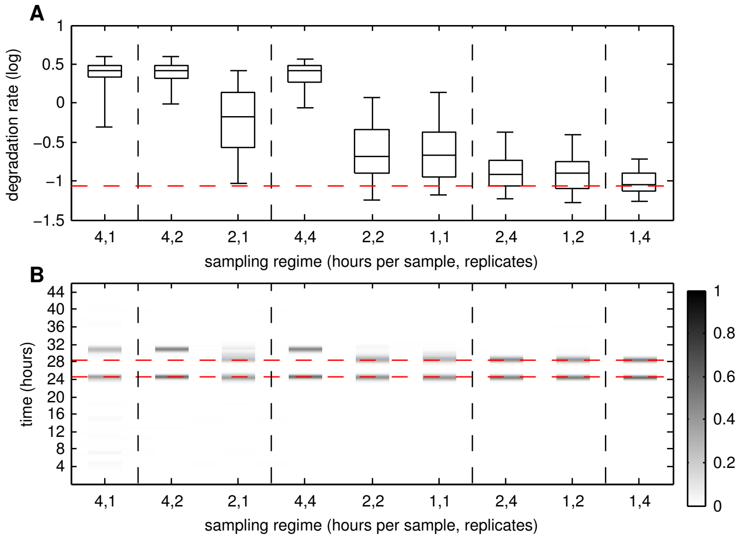

Supplementary Figure 15: Simulation study (sampling frequencies 1, 2 and 4h; Model SE6 with median noise level). Posterior distributions from switch event regime 6. **A**) distribution of estimated degradation rates (log scale) from 100 synthetic data sets for different combinations of (sampling frequency, number of replicates). Dashed red line shows the true degradation rate. **B**) Heatmap of the estimated switch using the Gaussian mixture model. Dashed red line shows true switch-time. Dashed vertical lines separate the sampling regimes by total sample size, in increasing order from 12 (far left) to 196 (far right).

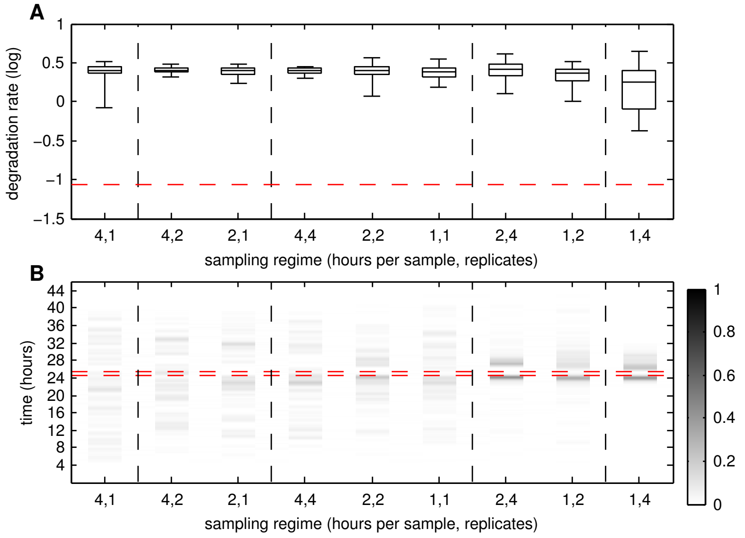

Supplementary Figure 16: Simulation study (sampling frequencies 1, 2 and 4h; Model SE4 with median noise level). Posterior distributions from switch event regime 4. **A**) distribution of estimated degradation rates (log scale) from 100 synthetic data sets for different combinations of (sampling frequency, number of replicates). Box plots have whiskers at 5th and 95th percentiles. Dashed red line shows the true degradation rate. **B**) Heatmap of the estimated switch using the Gaussian mixture model. Dashed red line shows true switch-time. Dashed vertical lines separate the sampling regimes by total sample size, in increasing order from 12 (far left) to 196 (far right). No sampling regimes are able to estimate parameters for the 2 switch events 1h apart accurately.

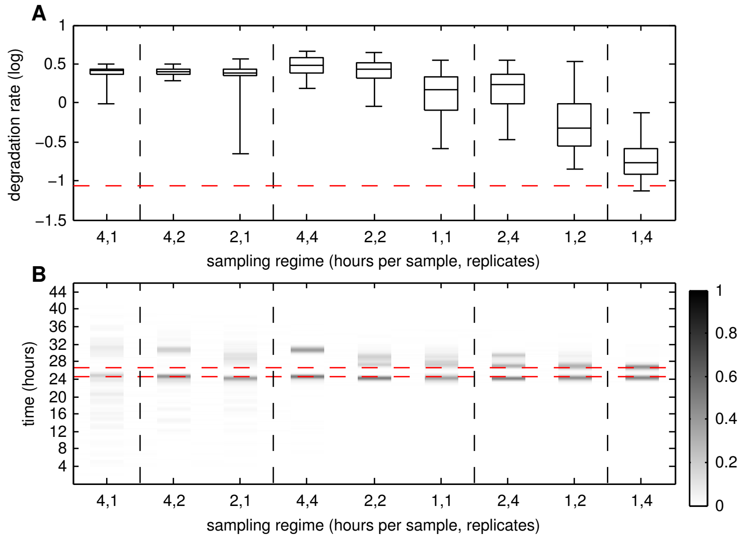

Supplementary Figure 17: Simulation study (sampling frequencies 1, 2 and 4h; Model SE5 with median noise level).Posterior distributions from switch event regime 5. **A**) distribution of estimated degradation rates (log scale) from 100 synthetic data sets for different combinations of (sampling frequency, number of replicates). Box plots have whiskers at 5th and 95th percentiles. Dashed red line shows the true degradation rate. **B**) Heatmap of the estimated switch using the Gaussian mixture model. Dashed red line shows true switch-time. Dashed vertical lines separate the sampling regimes by total sample size, in increasing order from 12 (far left) to 196 (far right). Sampling regimes with 1h observations are able to estimate parameters for the 2 switch events 2h apart.
